# Effect of receiving a customizable brochure on breast cancer patients' knowledge about their diagnosis and treatment: A randomized clinical trial

**DOI:** 10.1002/cam4.6215

**Published:** 2023-06-14

**Authors:** Cynthia Villarreal‐Garza, Ana S. Ferrigno, Cynthia De la Garza‐Ramos, Daniela Vazquez‐Juarez, Brizio Moreno‐Jaime, Yuly Remolina‐Bonilla, Manuel Segura‐Gonzalez, Ignacio Mariscal‐Ramirez, Florencia Perazzo, Georgina Garnica‐Jaliffe, Silvia Neciosup‐Delgado, Emilio Conde‐Flores, Shirly Mysler, Arlette Hernandez‐Ayala, Alondra Barajas‐Sanchez, Maria del Socorro Rios Mercado, Nelia Maria Noh‐Vazquez, Ricardo Garcia‐Rodriguez, Ana Platas, Jaime Tamez‐Salazar, Teresa Mireles‐Aguilar, Alejandra Platas

**Affiliations:** ^1^ Breast Cancer Center, Hospital Zambrano Hellion TecSalud Tecnologico de Monterrey San Pedro Garza Garcia Mexico; ^2^ Médicos e Investigadores en la Lucha contra el Cáncer de Mama Mexico City Mexico; ^3^ Hospital Regional del Instituto de Seguridad y Servicios Sociales de los Trabajadores del Estado (ISSSTE) Leon Mexico; ^4^ Hospital de Gineco‐Obstetricia No. 4 "Luis Castelazo Ayala" Instituto Mexicano del Seguro Social (IMSS) Mexico City Mexico; ^5^ Unidad Medica de Alta Especialidad del Instituto Mexicano del Seguro Social (IMSS) Merida Mexico; ^6^ Centro Médico Nacional de Occidente Instituto Mexicano del Seguro Social (IMSS) Guadalajara Mexico; ^7^ Section of Oncology CEMIC Buenos Aires Buenos Aires Argentina; ^8^ Hospital General de Mexico Mexico City Mexico; ^9^ Departamento de Medicina Oncologica Instituto Nacional de Enfermedades Neoplásicas Lima Peru; ^10^ Medical Oncology Research Unit Medica Sur Hospital and Clinical Foundation Mexico City Mexico

**Keywords:** breast cancer, Latin America, patient education, shared decision‐making, written information

## Abstract

**Background:**

Patients' lack of knowledge about their own disease may function as a barrier to shared decision‐making and well‐being. This study aimed to evaluate the impact of written educational materials on breast cancer patients.

**Methods:**

This multicenter, parallel, unblinded, randomized trial included Latin American women aged ≥18 years with a recent breast cancer diagnosis yet to start systemic therapy. Participants underwent randomization in a 1:1 ratio to receive a customizable or standard educational brochure. The primary objective was accurate identification of molecular subtype. Secondary objectives included identification of clinical stage, treatment options, participation in decision‐making, perceived quality of information received, and illness uncertainty. Follow‐up occurred at 7–21 and 30–51 days post‐randomization. ClinicalTrials.gov identifier: NCT05798312.

**Results:**

One hundred sixty‐five breast cancer patients with a median age of 53 years and 61 days from diagnosis were included (customizable: 82; standard: 83). At first available assessment, 52%, 48%, and 30% identified their molecular subtype, disease stage, and guideline‐endorsed systemic treatment strategy, respectively. Accurate molecular subtype and stage identification were similar between groups. Per multivariate analysis, customizable brochure recipients were more likely to identify their guideline‐recommended treatment modalities (OR: 4.20,*p* = 0.001). There were no differences between groups in the perceived quality of information received or illness uncertainty. Customizable brochure recipients reported increased participation in decision‐making (*p* = 0.042).

**Conclusions:**

Over one third of recently diagnosed breast cancer patients are incognizant of their disease characteristics and treatment options. This study demonstrates a need to improve patient education and shows that customizable educational materials increase patients' understanding of recommended systemic therapies according to individual breast cancer characteristics.

## 1 INTRODUCTION

Optimal treatment strategies and prognosis of breast cancer are largely dictated by three variables: molecular subtype, disease stage, and patient preferences.^1^ In clinical practice, active participation of patients in treatment decision‐making is not only desirable but expected. Nonetheless, the lack of patient knowledge about their disease, treatment options, and potential adverse events may act as a significant barrier to patient participation and ownership of their care.^2^, ^3^ Breast cancer patients' limited understanding of their diagnosis can have a substantial effect not only on the ability to participate in treatment decisions, but also on their emotional well‐being, perception of quality of care, and treatment adherence, all of which might indirectly impact clinical outcomes.^2^, ^4^, ^5^, ^6^, ^7^, ^8^, ^9^, ^10^ Furthermore, correct identification of tumor molecular subtype, based on the expression of hormone receptors and human epidermal growth factor receptor 2 (HER2), has been associated with receipt of guideline‐endorsed hormone therapy and chemotherapy, while correct identification of disease stage has been associated with receipt of recommended radiation therapy.^5^ This suggests that insufficient patient awareness of molecular subtype and stage may limit patient advocacy for their guideline‐recommended therapy and treatment adherence.

Limited data have been published regarding the knowledge level of breast cancer patients about their diagnosis. It has been reported that 13%–65% of patients do not know whether their tumor is hormone sensitive, 34%–70% are unaware of their HER2 status, and 28%–49% do not know their disease stage.^11^, ^12^, ^13^, ^14^, ^15^, ^16^, ^17^, ^18^ The proportion of patients that incorrectly describe their tumor characteristics (e.g., around 22% for disease stage in reported literature^12^, ^16^) implies that patients may not only ignore important details about their disease, but can also have an erroneous perception of their diagnosis that can further hinder shared decision‐making.

Previous studies have demonstrated the feasibility and utility of educational interventions to improve breast cancer patients' awareness of their disease.^17^, ^19^, ^20^ However, no previous studies have evaluated the level of knowledge about their disease in recently diagnosed, Latin American breast cancer patients or the impact of written educational materials on this population. The primary objective of this study was to evaluate whether a customizable educational brochure helped breast cancer patients better identify their molecular subtype compared to a standard (i.e., non‐customizable) educational brochure. Accurate identification of molecular subtype was chosen as the primary objective as hormone receptor and HER2 status are usually available to the medical oncologist when first determining the systemic treatment modalities for which a patient is eligible, leading to treatment discussions in the initial clinic visits. Secondary objectives included assessing whether a customizable brochure helped patients better identify their clinical stage and recommended systemic therapy modalities, improved patient participation in treatment decision‐making, and reduced illness uncertainty compared to a standard brochure.

## 2 MATERIALS AND METHODS

### 2.1 Patient selection

This study is a parallel‐arm, multicenter, international, unblinded, randomized clinical trial. Women aged ≥18 years with a recent breast cancer diagnosis (i.e., <6 months) who were yet to start systemic therapy (i.e., chemotherapy, hormone therapy, anti‐HER2 agents, and immunotherapy) were eligible to participate if they were scheduled for their first medical oncology appointment on which they would discuss their systemic therapy options. Exclusion criteria included known intellectual disability and inability to read or write.

Participant recruitment was undertaken in eight centers: Hospital Zambrano Hellion, San Pedro Garza Garcia, Mexico (05/2021–03/2022); Hospital Regional del Instituto de Seguridad y Servicios Sociales de los Trabajadores del Estado, Leon, Mexico (05–12/2021); Hospital de Gineco‐Obstetricia No. 4 “Luis Castelazo Ayala,” Mexico City, Mexico (10–12/2021); Unidad Medica de Alta Especialidad del Instituto Mexicano del Seguro Social, Merida, Mexico (08/2021–02/2022), Centro Médico Nacional de Occidente, Guadalajara, Mexico (11/2021–03/2022), Centro de Educación Medica e Investigaciones Clínicas “Norberto Quirno,” Buenos Aires, Argentina (01–03/2022), Hospital General de Mexico, Mexico City, Mexico (09/2021–02/2022), and Instituto Nacional de Enfermedades Neoplasicas, Lima, Peru (10–12/2021). All consecutive, eligible patients were invited to participate. Those who consented were randomized in a 1:1 ratio in blocks of two, four, or six patients using the Sealed Envelope website^21^ to receive either a customizable (Figure S1) or standard (Figure S2) educational brochure. One allocation sequence was generated per center to ensure a balanced randomization within each site. The sequence was generated centrally, and study coordinators were informed digitally to which group patients had been randomized to only after consenting each participant.

Sample size was calculated hypothesizing the proportion of patients that correctly identified their molecular subtype would be 20% greater in the customizable brochure group compared to the standard brochure group. A total of 194 participants were deemed necessary to detect a 20% difference with a power of 80% and an alpha of 0.05. Patient recruitment phase ended once the anticipated number of participants needed was achieved.

### 2.2 Educational brochures

Educational brochures were developed by a multidisciplinary team comprised of a medical oncologist and a surgeon specialized in breast cancer, two clinical researchers, and a group of graphic designers with experience in educational materials for oncologic patients. Team members performed a pilot of the customizable brochure in a total of 27 patients from four healthcare centers to test for comprehension and perceived utility. Patients' opinions and feedback were incorporated into the design and content of the brochures. All members of the team and two psychologists specialized in caring for cancer patients approved the final versions. Both brochures contain the same educational content, explaining in lay terms what is breast cancer *in situ* versus invasive; how are clinical stages defined; what are estrogen, progesterone, and HER2 receptors, and how they determine molecular subtype; and what treatment modalities patients should receive according to individual disease characteristics. The customizable brochure has blank circles on the right side of each item so that patients can mark their disease characteristics (e.g., clinical stage, molecular subtype, and recommended treatment strategies) and a legend that suggests the patient to discuss the brochure with her physician to correctly select what options apply to their case. These additional features in the customizable brochure provide patients with the opportunity to personalize the educational material by marking their own disease characteristics and encourage active communication with their physician. Lastly, both brochures cover complementary services (e.g., genetics, psychology, nutrition, and fertility counseling), patient rights, and a glossary of frequent medical terms they may encounter. To minimize the risk of contamination between groups (i.e., patients allocated to different brochures sharing them with each other if they realize they are different), the brochures look similar on the outside.

### 2.3 Follow‐up

Patients who consented to participate were asked to complete seven questionnaires either in‐person, by telephone, or email. Study coordinators were instructed not to suggest whether or how participants might use the brochure while answering study surveys. Three of the questionnaires were answered 7–21 days after having received the allocated brochure: (1) a multiple‐choice survey with two sections: sociodemographic and disease knowledge. The sociodemographic information included age, educational attainment, and employment status. The knowledge section asked patients to identify their clinical stage, molecular subtype, and appropriate systemic treatment options. All items in the knowledge section included “I don't know” as a possible answer; (2) a survey to evaluate participants' perception of the utility of their educational brochure on a four‐point Likert‐type scale; (3) the third survey was the European Health Literacy Survey Questionnaire—short version (HLS‐EU‐Q16) in Spanish to evaluate participants' understanding and communication of medical information.^22^ This survey consists of 16 items rated on a four‐point Likert‐type scale subsequently dichotomized as 0 (very difficult and difficult) and 1 (easy and very easy). Total score equals the sum of all items and is classified as: adequate (13–16), problematic (9–12), and inadequate (0–8).

The four remaining questionnaires were answered 30–51 days after brochure receipt: (1) the knowledge section of the first survey was repeated to evaluate information retention; (2) the second survey was the 9‐item Shared Decision‐Making Questionnaire (SDM‐Q‐9) developed by Kriston et al.,^23^ and adapted to Spanish by De las Cuevas et al.^24^ and is rated on a 0–5 Likert‐type scale. Total score is obtained by transforming the sum of all items into a scale from 0 to 100, with a higher score representing a higher degree of patient involvement in decision‐making; (3) the third survey was the European Organisation for Research and Treatment of Cancer Quality of Life Group information questionnaire (EORTC QLQ‐INFO 25), developed by Arraras et al.^25^, ^26^ and is scored on a 0–100 scale; and (4) the final survey was a 29‐item Spanish version of Mishel Uncertainty Illness Scale (MUIS),^27^ in which each item is scored on a five‐point Likert‐type scale. Total score is the sum of all items and patients are classified as having a low uncertainty level (<59), moderate (59–87), or high (>87). Lastly, clinical information was collected at the time of randomization directly from medical records by study coordinators at each site.

### 2.4 Statistical analysis

All analyses were performed using R statistical (version 4.1.0, R Project for Statistical Computing) and RStudio (version 1.4.1717, R Foundation for Statistical Computing) software. Descriptive statistics were undertaken using frequency and proportions for categorical variables, and median and 95% confidence interval (95% CI) for quantitative variables. Fisher's exact and Mann–Whitney *U*‐tests were used to explore differences according to intervention groups, as appropriate. Exact McNemar tests were used to evaluate differences between first and second knowledge assessments. Patients with undetermined stage or optimal treatment strategy at the time of randomization were excluded from analyses testing their knowledge on these areas. Logistic regression models were designed to evaluate variables associated with patients correctly identifying clinical stage, molecular subtype, and appropriate systemic treatment strategies at the first available assessment. The first available assessment was defined as the first knowledge test available for each case (i.e., 7–21 days post‐randomization for patients that completed the surveys at the first prespecified timepoint or 30–51 days post‐allocation for those that did not). Statistical significance was defined with a two‐tailed *p‐*value <0.05.

This trial was approved by the institutional review board of Escuela de Medicina del Instituto Tecnologico y de Estudios Superiores de Monterrey (protocol ID: P000329‐IMAP‐CM‐CEIC‐CR003) and was reviewed by appropriate authorities of each participating center. The protocol was conducted in accordance with The Code of Ethics of the World Medical Association and informed consent was obtained from each participant prior to inclusion. The complete study protocol is available from the corresponding author upon reasonable request.

## 3 RESULTS

### 3.1 Participant characteristics

**FIGURE 1:**
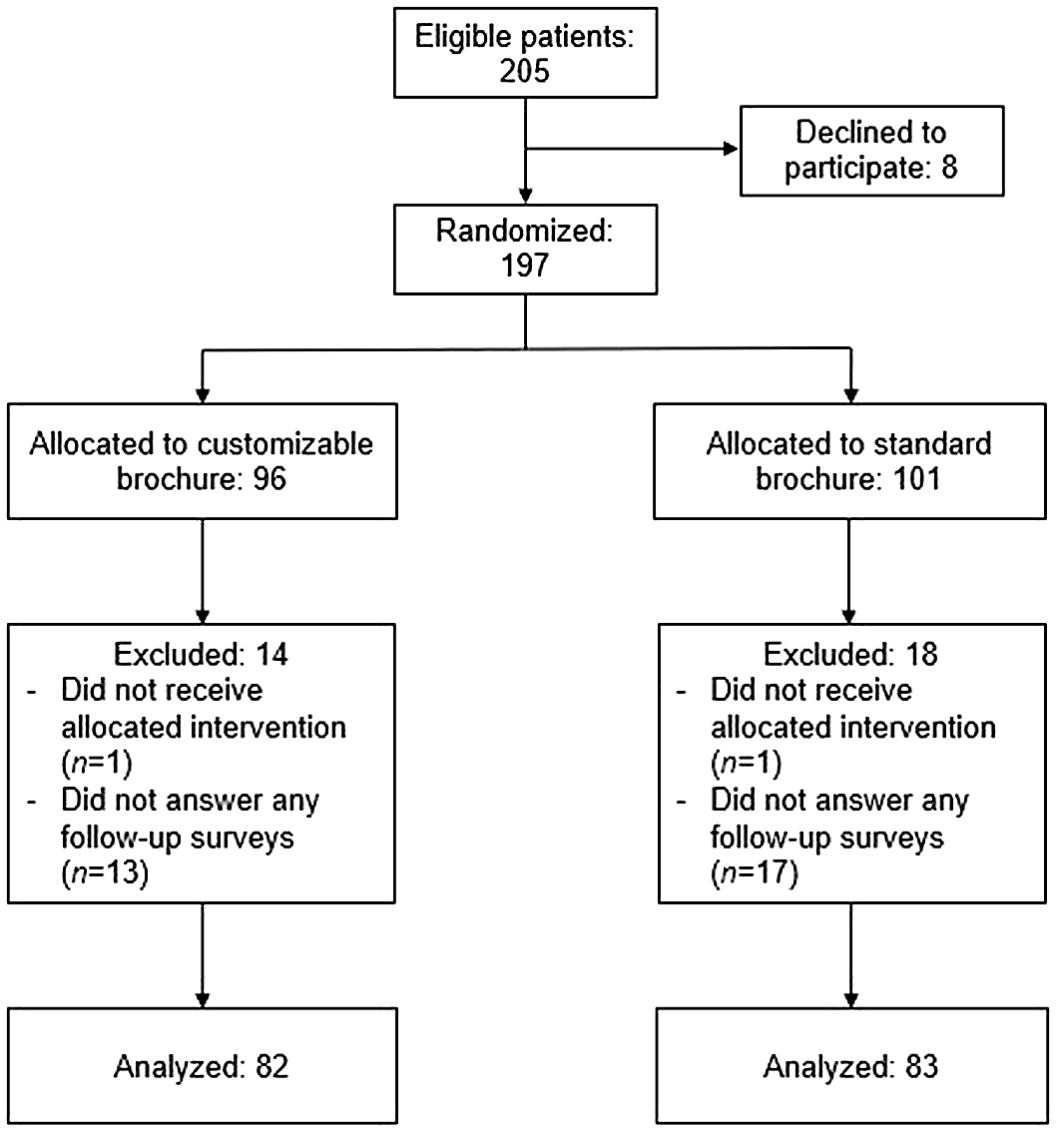
Participant flow diagram.

A total of 205 consecutive patients were eligible to participate (Figure 1). The final analysis included 165 participants (customizable brochure: 82 and standard brochure: 83). As there was no cross‐over, all analyses were performed according to the originally assigned groups.

Table 1 shows baseline participant characteristics. Overall, median age was 53 years (95% CI 49–55 years) at study inclusion. Most participants resided in Mexico (90.30%), completed at least post‐secondary education (64%), were currently employed (45%), had been diagnosed with early‐stage breast cancer (stages 0–IIIA: 86%), and were classified as HR+/HER2− disease (66%). Median time from breast cancer diagnosis to randomization was 61 days (95% CI 56–72.5).

**TABLE 1:** Sociodemographic and clinical characteristics of participants.

	Customizable brochure	Standard brochure	*p*‐value	Total
Country of residence				
Mexico	75 (91.46%)	74 (89.16%)	>0.999	149 (90.30%)
Argentina	5 (6.10%)	6 (7.23%)		11 (6.67%)
Peru	2 (2.44%)	3 (3.61%)		5 (3.03%)
Age at randomization				
Median (95% CI)	53 (49–59)	52 (48–57)	0.506	53 (49–55)
≤40 years	14 (17.07%)	13 (15.66%)	0.836	27 (16.36%)
>40 years	68 (82.93%)	70 (84.34%)		138 (83.64%)
Level of education				
None	2 (2.74%)	0	0.674	2 (1.40%)
Primary education	6 (8.22%)	8 (11.43%)		14 (9.79%)
Lower secondary education	12 (16.44%)	11 (15.71%)		23 (16.08%)
Upper secondary education	8 (10.96%)	4 (5.71%)		12 (8.39%)
Post‐secondary non‐tertiary education	14 (19.18%)	16 (22.86%)		30 (20.98%)
Tertiary education	31 (42.47%)	31 (44.29%)		62 (43.36%)
Occupation				
Work, full time	27 (36.99%)	24 (34.29%)	0.811	51 (35.66%)
Work, part time	5 (6.85%)	8 (11.43%)		13 (9.09%)
Incapacitated	3 (4.11%)	5 (7.14%)		8 (5.59%)
Retired	8 (10.96%)	7 (10.00%)		15 (10.49%)
Stay‐at‐home spouse	30 (41.10%)	26 (37.14%)		56 (39.16%)
Comorbidities				
No	35 (42.68%)	33 (40.24%)	0.874	68 (41.46%)
Yes	47 (57.32%)	49 (59.76%)		96 (58.54%)
Hypertension	20 (24.39%)	13 (15.85%)		34 (20.73%)
Thyroidopathy	5 (6.10%)	14 (17.07%)		18 (10.98%)
Diabetes mellitus	8 (9.76%)	8 (9.76%)		16 (9.76%)
Rheumatoid disease	3 (3.66%)	3 (3.66%)		6 (3.66%)
Cardiopathy	0	1 (1.22%)		1 (0.61%)
Pulmonary disease	1 (1.22%)	0		1 (0.61%)
Chronic renal disease	0	0		0
Obesity				
Yes	12 (14.63%)	7 (8.54%)	0.329	19 (11.59%)
No	70 (85.37%)	75 (91.46%)		145 (88.41%)
Health literacy according to HLS‐EU‐Q16				
Adequate	49 (70.00%)	43 (61.43%)	0.414	92 (65.71%)
Problematic	16 (22.86%)	18 (25.71%)		34 (24.29%)
Inadequate	5 (7.14%)	9 (12.86%)		14 (10.00%)
Time elapsed from breast cancer diagnosis to start of intervention (days)				
Median (95% CI)	67 (55–76.8)	58 (51.8–73.4)	0.243	61 (56–72.5)
Breast cancer stage at diagnosis				
Stage 0	1 (1.28%)	3 (3.90%)	0.890	4 (2.58%)
Stage IA	20 (25.64%)	23 (29.87%)		43 (27.74%)
Stage IB	1 (1.28%)	1 (1.30%)		2 (1.29%)
Stage IIA	17 (21.79%)	13 (16.88%)		30 (19.35%)
Stage IIB	15 (19.23%)	15 (19.48%)		30 (19.35%)
Stage IIIA	13 (16.67%)	11 (14.29%)		24 (15.48%)
Stage IIIB	4 (5.13%)	3 (3.90%)		7 (4.52%)
Stage IIIC	2 (2.56%)	5 (6.49%)		7 (4.52%)
Stage IV	5 (6.41%)	3 (3.90%)		8 (5.16%)
Molecular subtype				
HR+/HER2−	54 (69.23%)	49 (63.64%)	0.783	103 (66.45%)
HR+/HER2+	8 (10.26%)	11 (14.29%)		19 (12.26%)
HR−/HER2+	5 (6.41%)	7 (9.09%)		12 (7.74%)
HR−/HER2−	11 (14.10%)	10 (12.99%)		21 (13.55%)

*Note*: Missing values are not shown.

Abbreviations: HER2, human epidermal growth factor receptor 2; HLS‐EU‐Q16, European Health Literacy Survey Questionnaire, short version; HR, hormone receptor.

### 3.2 Participant knowledge regarding breast cancer

Participants' knowledge about their diagnosis and guideline‐recommended therapy was evaluated twice. A total of 141 patients completed the survey at the first prespecified timepoint after a median of 18 days (95% CI 15–19) since randomization and receipt of their corresponding brochure. One hundred forty‐nine participants completed the survey at the second‐prespecified timepoint after a median of 45 days (95% CI 42–48.7) from intervention allocation. At the first knowledge assessment available for each participant (median of 19 days [95% CI 18–20] post‐randomization), 75 of 157 respondents (48%) correctly identified their disease stage, 85 of 165 patients (52%) identified their molecular subtype, and 44 of 148 respondents (30%) accurately identified the systemic therapies they were eligible for according to their individual disease characteristics.

**FIGURE 2:**
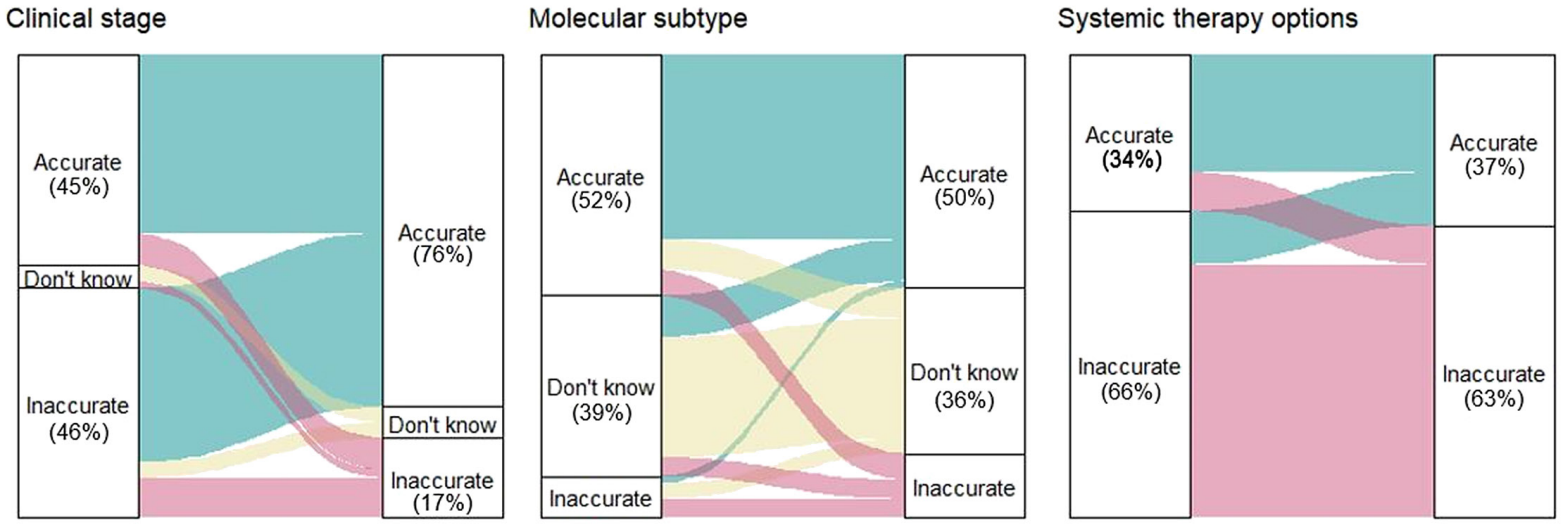
Proportion of patients that correctly identified their clinical stage, molecular subtype, and systemic treatment options according to their disease characteristics on the first (left) and second (right) assessment. The colored lines between assessments represent the change in response between assessments, weighted according to the proportion of participants. Represented in green are participants that answered accurately, in yellow those that responded “I don't know,” and in red those that answered inaccurately in the second assessment.

A total of 125 (76%) participants answered the knowledge survey at both prespecified follow‐up periods. Overall, the proportion of patients that correctly identified their clinical stage improved upon second assessment (45% vs. 76%, *p* < 0.001) (Figure 2). The proportion of patients that identified their molecular subtype and guideline‐endorsed systemic therapies did not differ between assessments (52% vs. 50%, *p =* 0.851 and 34% vs. 37%, *p* = 0.648, respectively).

Table 2 shows the proportion of respondents that correctly identified their disease characteristics and guideline‐endorsed systemic treatment strategies at each of the assessed timepoints according to the type of brochure received. At the first prespecified timepoint (i.e., 7–21 days post‐randomization), participants that received the customizable brochure were numerically more likely to correctly identify their progesterone receptor status (customizable: 79% vs. standard: 61%), HER2 status (customizable: 76% vs. standard: 59%), and overall molecular subtype (customizable: 56% vs. standard: 46%), although this did not reach statistical significance (*p* > 0.05). The proportion of respondents that accurately identified their disease stage was similar between groups (customizable: 43% vs. standard: 42%). Correct identification of guideline‐recommended systemic therapies was significantly higher in respondents who received the customizable brochure at first assessment (customizable: 43% vs. standard: 17%, *p* = 0.002). Those assigned to the customizable brochure were more likely to correctly identify whether they were eligible to receive hormone therapy (customizable: 79% vs. standard: 57%, *p =* 0.011) and immunotherapy (customizable: 56% vs. standard: 25%, *p* = 0.001), and demonstrated a non‐statistically significant tendency to improved recognition of need for radiotherapy (customizable: 70% vs. standard: 57%), and need for anti‐HER2 agents (customizable: 76% vs. standard: 59%). As shown in Table 2, similar results were obtained at the second prespecified timepoint.

**TABLE 2:** Participants' knowledge about their diagnosis and treatment options according to their disease characteristics at first and second assessment.

	Customizable brochure	Standard brochure	*p*‐value	Total
First assessment (7–21 days after randomization)				
Disease extent (invasive vs. in situ)				
Accurate	45 (63.38%)	32 (48.48%)	0.190	77 (56.20%)
Inaccurate	8 (11.27%)	12 (18.18%)		20 (14.60%)
Don't know	18 (25.35%)	22 (33.33%)		40 (29.20%)
Breast cancer stage (0, I, II, III, or IV)				
Accurate	29 (42.65%)	27 (41.54%)	>0.999	56 (42.11%)
Inaccurate	34 (50.00%)	33 (50.77%)		67 (50.38%)
Don't know	5 (7.35%)	5 (7.69%)		10 (7.52%)
Estrogen receptor status (positive vs. negative)				
Accurate	54 (76.06%)	55 (80.88%)	0.509	109 (78.42%)
Inaccurate	4 (5.63%)	1 (1.47%)		5 (3.60%)
Don't know	13 (18.31%)	12 (17.65%)		25 (17.99%)
Progesterone receptor status (positive vs. negative)				
Accurate	55 (78.57%)	42 (60.87%)	0.057	97 (69.78%)
Inaccurate	2 (2.86%)	6 (8.70%)		8 (5.76%)
Don't know	13 (18.57%)	21 (30.43%)		34 (24.46%)
HER2 status (amplified vs. not amplified)				
Accurate	54 (76.06%)	40 (58.82%)	0.056	94 (67.63%)
Inaccurate	1 (1.41%)	5 (7.35%)		6 (4.32%)
Don't know	16 (22.54%)	23 (33.82%)		39 (28.06%)
Molecular subtype (HR+/HER2−, HR+/HER2+, HR−/HER2+, or HR−/HER2−)				
Accurate	40 (55.56%)	32 (46.38%)	0.238	72 (51.06%)
Inaccurate	4 (5.56%)	9 (13.04%)		13 (9.22%)
Don't know	28 (38.89%)	28 (40.58%)		56 (39.72%)
Need for radiotherapy (yes vs. no)				
Accurate	40 (70.18%)	31 (57.41%)	0.394	71 (63.96%)
Inaccurate	2 (3.51%)	4 (7.41%)		6 (5.41%)
Don't know	15 (26.32%)	19 (35.19%)		34 (30.63%)
Need for chemotherapy (yes vs. no)				
Accurate	60 (92.31%)	58 (92.06%)	>0.999	118 (92.19%)
Inaccurate	1 (1.54%)	1 (1.59%)		2 (1.56%)
Don't know	4 (6.15%)	4 (6.35%)		8 (6.25%)
Chemotherapy timing (neoadjuvant only, adjuvant only, both, or never)				
Accurate	43 (66.15%)	44 (69.84%)	0.766	87 (67.97%)
Inaccurate	18 (27.69%)	14 (22.22%)		32 (25.00%)
Don't know	4 (6.15%)	5 (7.94%)		9 (7.03%)
Need for hormone therapy (yes vs. no)				
Accurate	57 (79.17%)	39 (56.52%)	**0.011**	96 (68.09%)
Inaccurate	3 (4.17%)	5 (5.80%)		7 (4.96%)
Don't know	12 (16.67%)	26 (37.68%)		38 (26.95%)
Need for anti‐HER2 therapy (yes vs. no)				
Accurate	54 (76.06%)	40 (58.82%)	0.056	94 (67.63%)
Inaccurate	1 (1.41%)	5 (7.35%)		6 (4.32%)
Don't know	16 (22.54%)	23 (33.82%)		39 (28.06%)
Need for immunotherapy (yes vs. no)				
Accurate	37 (56.06%)	15 (25.42%)	**0.001**	52 (41.60%)
Inaccurate	3 (4.55%)	3 (5.08%)		6 (4.80%)
Don't know	26 (39.39%)	41 (69.49%)		67 (53.60%)
Types of guideline‐endorsed systemic therapy according to their disease characteristics				
Accurate	26 (43.33%)	9 (16.67%)	**0.002**	35 (30.70%)
Inaccurate	34 (56.67%)	45 (83.33%)		79 (69.30%)
Used allocated brochure when answering the survey				
Yes	44 (61.11%)	38 (56.72%)	0.610	82 (58.99%)
No	28 (38.89%)	29 (43.28%)		57 (41.01%)
Second assessment (30–51 days after randomization)				
Disease extent (invasive vs. in situ)				
Accurate	45 (63.38%)	34 (48.57%)	0.223	79 (56.03%)
Inaccurate	14 (19.72%)	19 (27.14%)		33 (23.40%)
Don't know	12 (16.90%)	17 (24.29%)		29 (20.57%)
Breast cancer stage (0, I, II, III, or IV)				
Accurate	57 (80.28%)	51 (72.86%)	0.122	108 (76.60%)
Inaccurate	7 (9.86%)	15 (21.43%)		22 (15.60%)
Don't know	7 (9.86%)	4 (5.71%)		11 (7.80%)
Estrogen receptor status (positive vs. negative)				
Accurate	55 (76.39%)	53 (73.61%)	0.919	108 (75.00%)
Inaccurate	5 (6.94%)	6 (8.33%)		11 (7.64%)
Don't know	12 (16.67%)	13 (18.06%)		25 (17.36%)
Progesterone receptor status (positive vs. negative)				
Accurate	53 (72.60%)	43 (60.56%)	0.065	96 (66.67%)
Inaccurate	2 (2.74%)	9 (12.68%)		11 (7.64%)
Don't know	18 (24.66%)	19 (26.76%)		37 (25.69%)
HER2 status (amplified vs. not amplified)				
Accurate	56 (75.68%)	46 (63.89%)	0.305	102 (69.86%)
Inaccurate	5 (6.76%)	8 (11.11%)		13 (8.90%)
Don't know	13 (17.57%)	18 (25.00%)		31 (21.23%)
Molecular subtype (HR+/HER2−, HR+/HER2+, HR−/HER2+, or HR−/HER2−)				
Accurate	41 (55.41%)	34 (46.58%)	0.082	75 (51.02%)
Inaccurate	5 (6.76%)	14 (19.18%)		19 (12.93%)
Don't know	28 (37.84%)	25 (34.25%)		53 (36.05%)
Need for chemotherapy (yes vs. no)				
Accurate	62 (93.94%)	65 (92.86%)	0.663	127 (93.38%)
Inaccurate	2 (3.03%)	1 (1.43%)		3 (2.21%)
Don't know	2 (3.03%)	4 (5.71%)		6 (4.41%)
Chemotherapy timing (neoadjuvant only, adjuvant only, both, or never)				
Accurate	48 (87.27%)	51 (94.44%)	0.320	99 (90.83%)
Inaccurate	7 (12.73%)	3 (5.56%)		10 (9.17%)
Don't know	0	0		0
Need for hormone therapy (yes vs. no)				
Accurate	56 (75.68%)	47 (62.67%)	0.226	103 (69.13%)
Inaccurate	4 (5.41%)	6 (8.00%)		10 (6.71%)
Don't know	14 (18.92%)	22 (29.33%)		36 (24.16%)
Need for anti‐HER2 therapy (yes vs. no)				
Accurate	51 (68.92%)	41 (54.67%)	0.133	92 (61.74%)
Inaccurate	3 (4.05%)	8 (10.67%)		11 (7.38%)
Don't know	20 (27.03%)	26 (34.67%)		46 (30.87%)
Need for immunotherapy (yes vs. no)				
Accurate	35 (50.72%)	22 (31.43%)	**0.021**	57 (41.01%)
Inaccurate	7 (10.14%)	4 (5.71%)		11 (7.91%)
Don't know	27 (39.13%)	44 (62.86%)		71 (51.08%)
Types of guideline‐endorsed systemic therapy according to their disease characteristics				
Accurate	26 (40.63%)	18 (27.69%)	0.140	44 (34.11%)
Inaccurate	38 (59.38%)	47 (72.31%)		85 (65.89%)
Used allocated brochure when answering the survey				
Yes	38 (51.35%)	42 (56.00%)	0.624	80 (53.69%)
No	36 (48.65%)	33 (44.00%)		69 (46.31%)

*Note*: Missing values or non‐assessable responses are not shown.

Abbreviations: HER2, human epidermal growth factor receptor 2; HR, hormone receptor.

Bold values are statistically significant *p* < 0.05.

Univariate logistic regression analyses were performed to assess factors associated with identification of clinical stage, molecular subtype, and systemic therapy options per individual disease characteristics in the first knowledge survey available (Table 3). None of the studied variables were associated with correct clinical stage identification. Participants from private healthcare institutions and those aged ≤40 years were more likely to correctly identify their tumor molecular subtype. However, in a multivariate model including the type of health insurance and age group, only the latter remained statistically associated with higher odds of identifying molecular subtype (odds ratio 1.88 [95% CI 0.96–3.66] and 4.98 [95% CI 1.77–14.02], respectively). In terms of correctly identifying their guideline‐endorsed systemic therapy, receiving a customizable brochure was the only variable statistically associated per univariate analysis. The odds ratio of correctly identifying systemic therapy options according to their diagnosis in participants who received a customizable brochure compared to those who received a standard brochure was 4.20 (95% CI 1.74–10.17, *p* = 0.001) per multivariate analysis after adjusting for age group, type of health insurance, and health literacy level.

**TABLE 3:** Univariate logistic regression analyses.

	Correct stage identification		Correct molecular subtype identification		Correct identification of systemic treatment options	
Variable	OR (95% CI)	*p*‐value	OR (95% CI)	*p*‐value	OR (95% CI)	*p‐*value
Country (other vs. Mexico)	0.33 (0.10–1.07)	0.064	2.23 (0.74–6.73)	0.155	0.16 (0.02–1.29)	0.085
Type of healthcare system (private vs. public)	1.22 (0.64–2.32)	0.542	2.00 (1.05–3.82)	**0.035**	0.84 (0.41–1.75)	0.647
Age (≤40 years vs. > 40 years)	0.85 (0.37–1.96)	0.704	5.23 (1.88–14.63)	**0.002**	1.74 (0.72–4.26)	0.221
Educational attainment (≥high school vs. < high school)	1.05 (0.50–2.24)	0.894	1.45 (0.69–3.05)	0.323	0.93 (0.39–2.21)	0.872
Occupational status (employed vs. other)	0.63 (0.31–1.26)	0.192	1.09 (0.57–2.11)	0.794	0.71 (0.32–1.56)	0.394
Comorbidities (yes vs. no)	0.66 (0.35–1.25)	0.201	0.93 (0.50–1.73)	0.810	0.72 (0.36–1.48)	0.374
Health literacy (adequate vs. problematic/inadequate)	1.36 (0.65–2.82)	0.413	1.35 (0.67–4.54)	0.405	1.67 (0.70–4.00)	0.247
Brochure received (customizable vs. not customizable)	0.84 (0.45–1.57)	0.579	1.44 (0.78–2.66)	0.242	3.52 (1.65–7.50)	**0.001**
Brochure consulted while answering the survey (yes vs. no)	0.62 (0.33–1.17)	0.141	1.23 (0.67–2.28)	0.507	1.27 (0.62–2.58)	0.515

Abbreviation: OR, odds ratio.

Bold values are statistically significant *p* < 0.05.

### 3.3 Participant satisfaction with the educational brochure

In terms of satisfaction with the brochure received, the only statistically significant difference observed was in perception that the brochure helped patients participate in treatment decisions (patients who selected “strongly agree” in the customizable brochure group: 60% vs. standard: 42%, *p* = 0.042) (Table 4). However, median scores of the SDM‐Q9 questionnaire did not differ between intervention groups (84.45 [95% CI 77.8–91.7] vs. 82.2 [95% CI 79.7–91.1], *p =* 0.656). Overall, 73% of participants reported that it was very necessary for patients to receive an informational brochure like the one they had received and 56% found it to be a very useful resource.

**TABLE 4:** Participant satisfaction with the educational brochure received.

	Customizable brochure	Standard brochure	*p*‐value	Total
Did you like receiving a brochure about your disease?				
Strongly agree	49 (70.00%)	43 (62.32%)	0.373	92 (66.19%)
Moderately agree	17 (24.29%)	23 (33.33%)		40 (28.78%)
Slightly agree	3 (4.29%)	1 (1.45%)		4 (2.88%)
Disagree	1 (1.43%)	2 (2.90%)		3 (2.16%)
How likely is it that you will keep the brochure and take it with you to future medical appointments?				
Very likely	49 (70.00%)	39 (56.52%)	0.115	88 (63.31%)
Moderately likely	15 (21.43%)	18 (26.09%)		33 (23.74%)
Slightly likely	4 (5.71%)	10 (14.49%)		14 (10.07%)
Not at all likely	2 (2.86%)	2 (2.90%)		4 (2.88%)
Did the brochure help you understand the characteristics of your disease?				
Strongly agree	40 (57.14%)	41 (59.42%)	0.864	81 (58.27%)
Moderately agree	24 (34.29%)	19 (27.54%)		43 (30.94%)
Slightly agree	6 (8.57%)	8 (11.59%)		14 (10.07%)
Disagree	0	1 (1.45%)		1 (0.72%)
Did the brochure help you remember your treatment options?				
Strongly agree	43 (61.43%)	34 (49.28%)	0.174	77 (55.40%)
Moderately agree	22 (31.43%)	28 (40.58%)		50 (35.97%)
Slightly agree	5 (7.14%)	5 (7.25%)		10 (7.19%)
Disagree	0	2 (2.90%)		2 (1.44%)
Did the brochure help you understand the information provided by the medical team?				
Strongly agree	41 (58.57%)	33 (47.83%)	0.236	74 (53.24%)
Moderately agree	25 (35.71%)	28 (40.58%)		53 (38.13%)
Slightly agree	3 (4.29%)	7 (10.14%)		10 (7.19%)
Disagree	1 (1.43%)	1 (1.45%)		2 (1.44%)
Did the brochure help you remember the information provided by the medical team?				
Strongly agree	38 (54.29%)	34 (49.28%)	0.612	72 (51.80%)
Moderately agree	25 (35.71%)	27 (39.13%)		52 (37.41%)
Slightly agree	6 (8.57%)	6 (8.70%)		12 (8.63%)
Disagree	1 (1.43%)	2 (2.90%)		3 (2.16%)
Overall, how useful did you find the brochure?				
Very useful	40 (57.14%)	38 (55.07%)	0.865	78 (56.12%)
Moderately useful	26 (37.14%)	26 (37.68%)		52 (37.41%)
Slightly useful	4 (5.71%)	4 (5.8%)		8 (5.76%)
Not at all useful	0	1 (1.45%)		1 (0.72%)
How useful has the brochure been when making treatment decisions with your physician?				
Very useful	33 (47.14%)	26 (37.68%)	0.304	59 (42.45%)
Moderately useful	29 (41.43%)	26 (37.68%)		55 (39.57%)
Slightly useful	4 (5.71%)	13 (18.84%)		17 (12.23%)
Not at all useful	4 (5.71%)	4 (4.80%)		8 (5.76%)
Did the brochure help you participate in the treatment decision‐making process?				
Strongly agree	42 (60.00%)	29 (42.03%)	**0.042**	71 (51.08%)
Moderately agree	17 (24.29%)	24 (34.78%)		41 (29.50%)
Slightly agree	5 (7.14%)	9 (13.04%)		14 (10.07%)
Disagree	6 (8.57%)	7 (10.14%)		13 (9.35%)
How likely is it that you will recommend the brochure you were given to other patients with your disease?				
Very likely	51 (72.86%)	45 (65.22%)	0.369	96 (69.06%)
Moderately likely	15 (21.43%)	22 (31.88%)		37 (26.62%)
Slightly likely	4 (5.71%)	1 (1.45%)		5 (3.60%)
Not at all likely	0	1 (1.45%)		1 (0.72%)
In your opinion, how necessary is it that every patient is given a brochure like the one you received?				
Very necessary	53 (75.71%)	49 (71.01%)	0.569	102 (73.38%)
Moderately necessary	13 (18.57%)	19 (27.54%)		32 (23.02%)
Slightly necessary	4 (5.71%)	1 (1.45%)		5 (3.60%)
Not at all necessary	0	0		0

*Note*: *p*‐values were calculated using dichotomized answers (strongly/very vs. all other responses).

A total of 141 participants answered the QLQ‐INFO25 questionnaire. The global score was similar in patients that received a customizable brochure and those who received a standard brochure (median score 55.6 [95% CI 48.5–58.4] vs. 54.7 [95% CI 48.4–56.5], *p* = 0.895). Likewise, subscale scores for information about disease (median score 66.7 [95% CI 50–66.7] vs. 58.3 [95% CI 51.5–66.7], *p* = 0.895), information about treatments (median score 60 [95% CI 53.3–66.7] vs. 60 [95% CI 53.3–66.7], *p* = 0.650), satisfaction with information received (median score 66.7 [95% CI 66.7–66.7] vs. 66.7 [95% CI 66.7–66.7], *p* = 0.879), and overall, if the information provided has been helpful (median score 66.7 [95% CI 66.7–66.7] vs. 66.7 [95% CI 66.7–66.7], *p* = 0.860) were not statistically different between intervention groups. Only one patient responded she wished to have received less information. In contrast, 101 (72%) respondents reported they wished to receive more information, including causes of breast cancer, symptoms, disease prognosis, more details about their disease and treatment strategies, nutrition, how to lead a healthy lifestyle, breast reconstruction, sexuality, psychological impact of disease (e.g., uncertainty, stress, self‐esteem, depression), support groups available, and disease surveillance strategies.

In terms of illness uncertainty, there was no difference according to the brochure received (median score 67 [95% CI 62–74] in the customizable group vs. 73 [95% CI 66.4–76.8] in the standard group, *p* = 0.280). Of 141 respondents of the MUIS survey, 26 (18%) were classified as having a high level of uncertainty, 80 (57%) moderate, and 35 (25%) a low level.

## 4 DISCUSSION

In this study, approximately half of recently diagnosed, Latin American breast cancer patients were able to correctly identify their disease stage or molecular subtype. Furthermore, only one third of participants identified which systemic treatments were recommended according to their individual tumor characteristics. These findings underscore the need to improve information provision and promote breast cancer patient education. In the research protocol, a customizable brochure was hypothesized to result in improved identification of molecular subtype by encouraging a dialog between patients and their treating physicians when identifying the options applicable to each case. However, no statistical differences were observed in patient knowledge about their molecular subtype or clinical stage at diagnosis according to the type of educational brochure received. Therefore, the hypothesis that the proportion of patients who would identify their molecular subtype with the customizable brochure would be 20% greater than the standard group was not met. Notably, patients allocated to the customizable brochure were significantly more likely to identify which systemic therapy they should receive according to their disease characteristics and perceived a stronger agreement that the brochure helped them participate in treatment decisions. This suggests that personalizing written materials was an effective tactic to enhance their educational value, possibly by stimulating shared‐decision making between patients and their treating physicians.

Previous studies have demonstrated that patient education interventions can enhance breast cancer patients' knowledge and comprehension. Buscemi et al. developed a culturally tailored smartphone application for Hispanic patients and found that its use was associated with a significant improvement in general breast cancer knowledge in this population.^19^ Nissen et al. found that breast cancer survivors' knowledge about their disease improved after receiving a treatment summary, reporting an increase of 12%, 9%, and 15% in the proportion of patients that correctly identified their disease stage, estrogen receptor status, and progesterone receptor status, respectively, after the intervention.^20^ Similarly, Ulloa et al. demonstrated that receiving a customizable card including disease stage, treatment received, and recurrence symptoms resulted in a 27% increase in the proportion of breast cancer survivors that correctly identified their stage on a follow‐up survey.^17^ Despite the proven feasibility and utility of patient education interventions, most published studies have focused on breast cancer survivors rather than patients about to begin treatment. Recently diagnosed patients represent a particularly vulnerable group as initial shock and distress can act as a barrier to understanding information about their disease and hinder participation in shared decision‐making.^2^

Patient knowledge regarding individual characteristics of their breast cancer can improve their understanding on recommended treatment strategies (e.g., hormone therapy in cases of hormone‐sensitive disease or trastuzumab in cases with HER2 amplification), which can lead to guideline‐endorsed treatment decisions and greater therapeutic adherence.^5^ Furthermore, providing patients with an adequate level of medical information about their disease has been associated with better satisfaction with care received and quality of life.^25^ Specifically, providing enough information about their disease can mitigate emotional distress in cancer patients by reducing illness uncertainty.^28^, ^29^ In this study, a high proportion of respondents strongly or moderately agreed the brochure they received had helped them understand the characteristics of their disease, remember information provided by the medical team, and participate in treatment decision‐making. Over two‐thirds of respondents thought it was very necessary for every breast cancer patient to receive a brochure like the one they received. This finding emphasizes the value of providing written information about their disease to recently diagnosed breast cancer patients. However, study participants expressed suboptimal provision of information and satisfaction (EORTC‐INFO‐25 median score = 55) and moderate levels of uncertainty (MUIS median = 69) irrespective of the type of brochure received, underscoring gaps in information needs of the population studied. Overall, 72% expressed a need for additional information, mainly in areas of disease causes, treatment‐related adverse events, self‐care, and available support services. This finding highlights the value of patients' participation in the design of educational materials to include information that may be of particular interest to them. Finally, a relatively high involvement in shared decision‐making was reported (SDM‐Q‐9 median score = 83), suggesting that Latin American breast cancer patients might be participating in care decision‐making with insufficient knowledge about their disease and consequences of their decisions.

This study has limitations to consider when interpreting the results. First, the study is underpowered to detect subtle differences according to the type of brochure received. Second, no multiple comparison correction was undertaken. Third, the sample studied is not representative of all Latin America and the results cannot be generalized. Fourth, because of the nature of the intervention, blinding of participants and treating physicians was not possible. Fifth, clinicians at participating centers were aware that patient knowledge was being assessed and it is possible that they might have been more inclined to emphasize disease characteristics. Sixth, due to participant attrition and nonresponse throughout the protocol, nonresponder bias cannot be excluded. Seventh, improvement in knowledge about their disease directly after receiving each of the brochures was not assessed as no pre‐intervention questionnaires were applied. Eight, patients assigned to the customizable educational brochure were encouraged to personalize their material with the assistance of healthcare providers, but mandatory discussion of brochures with the treating physicians was not enforced. Hence, patients assigned to customizable brochures might not have filled out the material or might have done so incorrectly if they did not discuss the contents with a physician, potentially underestimating the true utility of this type of written educational material.

Although patients were not involved in the development of the content of the educational material, they were interviewed to identify whether the vocabulary employed in the brochures was comprehensible and to evaluate the general perception of its utility. Their feedback and comments were incorporated into the final version of the brochure. Noteworthy, the purpose of this project was not to propose this brochure as a standard to be widely used, but to demonstrate that a customizable, educational material aids patients. The results are in line with this objective and affirm the usefulness of providing customizable, educational materials to patients.

## 5 CONCLUSION

In conclusion, a high proportion of recently diagnosed Latin American breast cancer patients remain oblivious to tumor characteristics such as molecular subtype and disease stage despite having received written educational materials. Both a customizable educational brochure and a standard, non‐customizable brochure were perceived as useful by participants in understanding their own disease. Nonetheless, most participants expressed a need for additional information and moderate‐to‐high levels of uncertainty, underscoring the need for improved information delivery strategies and the development of patient‐centered education materials that can address the unmet information needs of Latin American breast cancer patients. Notably, patients that received a customizable brochure were more likely to correctly identify which systemic treatment modalities they should receive according to their tumor characteristics. This finding indicates that customizable brochures improved patients' understanding of the recommended treatment modalities and could be an effective strategy to implement in clinical practice.

## AUTHOR CONTRIBUTIONS

**Cynthia Villarreal‐Garza:** Conceptualization (lead); data curation (equal); formal analysis (equal); funding acquisition (lead); investigation (lead); methodology (equal); project administration (lead); resources (equal); software (equal); supervision (lead); validation (supporting); visualization (equal); writing – original draft (supporting); writing – review and editing (supporting). **Ana Sofia Ferrigno Guajardo:** Conceptualization (lead); data curation (lead); formal analysis (lead); funding acquisition (supporting); investigation (lead); methodology (lead); project administration (lead); resources (supporting); software (equal); supervision (lead); validation (equal); visualization (equal); writing – original draft (lead); writing – review and editing (lead). **Cynthia De la Garza‐Ramos:** Conceptualization (lead); data curation (equal); formal analysis (equal); funding acquisition (supporting); investigation (supporting); methodology (equal); project administration (supporting); resources (supporting); software (supporting); supervision (supporting); validation (supporting); visualization (supporting); writing – original draft (lead); writing – review and editing (lead). **Daniela Vazquez‐Juarez:** Investigation (equal); methodology (equal); project administration (equal); resources (equal); supervision (equal); validation (equal); writing – original draft (supporting); writing – review and editing (equal). **Brizio Moreno‐Jaime:** Funding acquisition (equal); investigation (equal); methodology (equal); project administration (equal); resources (equal); supervision (equal); writing – review and editing (equal). **Yuly Andrea Remolina Bonilla:** Funding acquisition (equal); investigation (equal); methodology (equal); project administration (equal); resources (equal); supervision (equal); writing – review and editing (equal). **Manuel Segura‐Gonzalez:** Investigation (equal); methodology (equal); project administration (equal); resources (equal); supervision (equal); writing – review and editing (equal). **Ignacio Mariscal‐Ramirez:** Funding acquisition (equal); investigation (equal); methodology (equal); project administration (equal); resources (equal); supervision (equal); writing – review and editing (equal). **Florencia Perazzo:** Funding acquisition (equal); investigation (equal); methodology (equal); project administration (equal); resources (equal); supervision (equal); visualization (equal); writing – review and editing (equal). **Georgina Garnica‐Jaliffe:** Funding acquisition (equal); investigation (equal); methodology (equal); project administration (equal); resources (equal); software (equal); supervision (equal); writing – review and editing (equal). **Silvia Neciosup‐Delgado:** Funding acquisition (equal); investigation (equal); methodology (equal); project administration (equal); resources (equal); supervision (equal); writing – review and editing (equal). **Emilio Conde‐Flores:** Data curation (equal); investigation (equal); methodology (equal); project administration (equal); resources (equal); supervision (equal); writing – review and editing (equal). **Shirly Judith Mysler:** Data curation (equal); investigation (equal); methodology (equal); project administration (equal); resources (equal); visualization (equal); writing – review and editing (equal). **Arlette Hernandez‐Ayala:** Data curation (equal); investigation (equal); project administration (equal); resources (equal); software (equal); writing – review and editing (equal). **Alondra Barajas‐Sanchez:** Data curation (equal); investigation (equal); methodology (equal); resources (equal); supervision (equal); visualization (equal); writing – review and editing (equal). **Maria del Socorro Rios Mercado:** Data curation (equal); investigation (equal); methodology (equal); project administration (equal); resources (equal); supervision (equal); writing – review and editing (equal). **Nelia Maria Noh‐Vazquez:** Data curation (equal); methodology (equal); project administration (equal); resources (equal); supervision (equal); visualization (equal); writing – review and editing (equal). **Ricardo Garcia‐Rodriguez:** Data curation (equal); investigation (equal); methodology (equal); project administration (equal); resources (equal); writing – review and editing (equal). **Ana Platas:** Data curation (equal); investigation (equal); methodology (equal); project administration (equal); resources (equal); supervision (equal); validation (equal); writing – review and editing (equal). **Jaime Tamez‐Salazar:** Funding acquisition (equal); investigation (equal); methodology (equal); project administration (equal); resources (equal); supervision (equal); writing – review and editing (equal). **Teresa Mireles‐Aguilar:** Funding acquisition (equal); investigation (equal); methodology (equal); project administration (equal); resources (equal); supervision (equal); writing – review and editing (equal). **Alejandra Platas:** Data curation (equal); project administration (equal); resources (equal); software (equal); supervision (equal); validation (equal); visualization (equal); writing – review and editing (equal).

## FUNDING INFORMATION

This work was supported by Roche Mexico.

## CONFLICT OF INTEREST STATEMENT

C.V.G: Reports grants, personal fees, and non‐financial support from AstraZeneca; grants, personal fees, and non‐financial support from Roche; personal fees and non‐financial support from MSD Oncology; personal fees and non‐financial support from Pfizer; non‐financial support from Novartis; and personal fees from Eli Lilly outside the submitted work. A.S.F: Reports consulting with Roche Mexico. S.N.D: Reports consultancy role for AstraZeneca, Genentech, GlaxoSmithKline, Medscape, Merck‐Sharp, Novartis, Pfizer, Roche, Janssen; institutional financial support from Amgen, AstraZeneca, Bristol‐Myers Squibb, Daiichi, Novartis, Merck, Millenium, Pfizer, Roche, Pfizer; being Chair for ABC Global Alliance and ABC Consensus. M.S.G: Reports honoraria from Roche, ESAI, AMGEN. Y.R.B: Reports support for meetings or travel from Bristol‐Myers Squibb and Bayer. J.T.S: Reports grant support and honoraria from Roche Mexico. Other authors: No disclosures.

## Supporting information


Figure S1.

Figure S2.

Figure S3.

## Data Availability

N/A.
