# Supplementary material for: Effect of receiving a customizable brochure on breast cancer patients' knowledge about their diagnosis and treatment: A randomized clinical trial
Source: Cancer Med. 2023 Jun 14;12(14):15612–27. doi: 10.1002/cam4.6215 (PMC10417173; doi:10.1002/cam4.6215)
Supplement: Supplementary file 1 — Figure S1. [file CAM4-12-15612-s001.pdf]

CONOCIENDO MI CÁNCER DE MAMA

ALERTA ROSA

Yo soy ______________________

Te sugerimos contestar junto con tu médico esta guía para conocer mejor tu cáncer y tus opciones de tratamiento.

Rellena ✓

Conociendo mi cáncer de mama

Es una enfermedad causada por el crecimiento anormal de células de la mama que forman un tumor maligno

¿Qué tipo de cáncer de mama tengo?

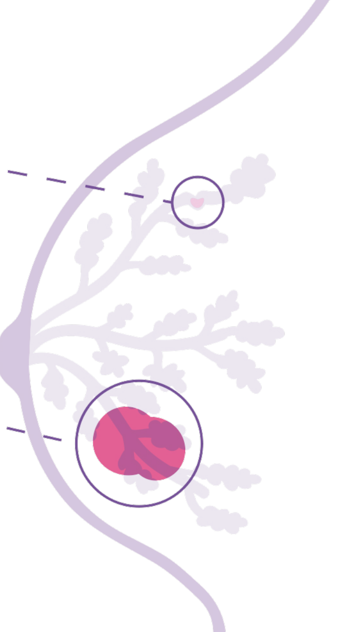

○ In situ

Las células malignas se localizan dentro de los ductos que conducen la leche hasta el pezón

○ Invasor

Las células malignas crecen fuera de los ductos e invaden otras partes de la mama

¿En qué etapa está mi cáncer?

○ Etapa 0

Cáncer in situ

○ Etapa 1

Tumor menor de 2 cm y no ha afectado a los ganglios de la axila

○ Etapa 2

Tumor de 2 a 5 cm y no ha afectado a los ganglios de la axila o sólo ha afectado 1 a 3 ganglios

○ Etapa 3

Tumor mayor a 5 cm o que ha afectado 4 o más ganglios

○ Etapa 4

El tumor se ha extendido a otros órganos (cáncer metastásico)

¿Qué receptores hacen crecer mi cáncer?

Receptor de Estrógeno

○ Positivo, ya que el tumor depende de estrógeno para su crecimiento

○ Negativo

Receptor de Progesterona

○ Positivo, ya que el tumor depende de progesterona para su crecimiento

○ Negativo

HER2

○ Positivo, ya que el tumor está sobrecargado de receptores HER2

○ Negativo

○ Si los tres son negativos, mi tipo de cáncer de mama es triple negativo

Conociendo mis opciones de tratamiento

1. ¿Soy candidata para cirugía de mama?

○ Sí

○ No

2. ¿Qué tipo de cirugía necesito en mi mama? ○ Por definir

○ Cirugía conservadora — Mi tumor es pequeño y está localizado en una sola parte de la mama

○ Mastectomía — ● Con reconstrucción ● Sin reconstrucción

3. ¿Cuál es la cirugía que necesito para mi axila? ○ Por definir

○ Ganglio centinela — Mis ganglios parecen que no están afectados

○ Disección radical axilar — Mis ganglios están afectados

4. ¿Debo recibir radioterapia?

○ Sí, porque: ○ No

● Me realizaron una cirugía conservadora

● Mi tumor mide más de 5 cm

● Tengo ganglios positivos

5. ¿Soy candidata a recibir quimioterapia?

○ Sí ○ No ○ Por definir

○ Antes de la cirugía:

● Tengo cáncer etapa 3

● Tengo cáncer etapa 2 y es HER2 positivo o triple negativo

○ Después de la cirugía

6. ¿Debo recibir tratamiento anti-HER2?

○ Sí, porque: ○ No

Mi cáncer es HER2 positivo

7. ¿Soy candidata a tomar tratamiento anti-hormonal?

○ Sí, porque: ○ No

Mi cáncer tiene el receptor de estrógeno y/o progesterona positivo

8. ¿Debo recibir tratamiento con inmunoterapia?

○ Sí, porque: ○ No

Tengo cáncer etapa 4, triple negativo, PD-L1 positivo

○ Por definir

9. ¿Necesito realizar una prueba genética para saber si mi cáncer es hereditario?

○ Sí, porque: ○ No

Tengo factores de riesgo para cáncer hereditario

10. ¿Que servicios complementarios requiero?

○ Genética ○ Psicología ○ Nutrición

○ Preservación de la fertilidad ○ Asesoramiento en sexualidad ○ Cuidados de soporte o paliativos

○ Grupos de apoyo ○ Terapia de linfedema

Glosario

Cirugía de mama

Cirugía conservadora: Se retira el tumor con un margen de tejido sano para dejar el resto de la mama libre de cáncer. Siempre se debe acompañar de radioterapia.

Mastectomía: Se retira toda la mama.

Cirugía de axila

Ganglio centinela: Es el primer ganglio al que las células malignas tienen más probabilidad de invadir. Si es negativo, no hay necesidad de quitar el resto de los ganglios de la axila.

Disección radical axilar: Es un procedimiento donde se quitan los ganglios de la axila cuando están afectados.

Tratamientos

Radioterapia: Se utiliza radiación en la mama y/o axila para destruir las células malignas y así reducir las probabilidades de que el cáncer regrese al sitio en donde se originó.

Quimioterapia: Son medicamentos que se administran por la vena para destruir las células malignas en cualquier parte del cuerpo.

Tratamiento anti-HER2 (trastuzumab y pertuzumab): Son medicamentos específicos (anticuerpos) que atacan células que tienen receptores HER2 positivos.

Tratamiento anti-hormonal (tamoxifeno, anastrozol, letrozol, exemestano, gosereline, leuprolide o triptoreline): Son medicamentos que detienen el crecimiento de las células malignas cuando tienen receptores hormonales positivos.

Inmunoterapia (atezolizumab): Son medicamentos que activan el sistema inmune para atacar al cáncer. Actualmente solo se emplea en el cáncer de mama triple negativo etapa 4 que tiene un receptor especial llamado PD-L1.

Cáncer hereditario

Algunos factores de riesgo para cáncer hereditario:

- Tú o alguien de tu familia fue diagnosticado con cáncer de mama antes de cumplir 50 años.
- Hay varias personas con cáncer de mama y/o de ovario en tu familia.
- Tienes cáncer de mama triple negativo.
- Hay antecedente de otros tipos de cáncer en varios miembros de tu familia.
- Tú o algún familiar tuyo ha tenido cáncer de mama en ambos senos.
- Hay algún hombre en la familia que ha sido diagnosticado con cáncer de mama.

Es importante que conozcas tus derechos como paciente

- Tener acceso oportuno a servicios de calidad para diagnóstico y tratamiento.
- Recibir atención médica adecuada, trato digno y respetuoso.
- Recibir información suficiente, clara y veraz.
- Decidir libremente tu tratamiento.
- Contar con facilidades para pedir una segunda opinión.

¡Haz valer tus derechos!

ALERTA ROSA

@alertarosamx

@alertarosamx

www.alertarosa.com
