# Supplementary material for: Effect of receiving a customizable brochure on breast cancer patients' knowledge about their diagnosis and treatment: A randomized clinical trial
Source: Cancer Med. 2023 Jun 14;12(14):15612–27. doi: 10.1002/cam4.6215 (PMC10417173; doi:10.1002/cam4.6215)
Supplement: Supplementary file 2 — Figure S2. [file CAM4-12-15612-s002.pdf]

CONOCIENDO MI CÁNCER DE MAMA

ALERTA ROSA

Conociendo el cáncer de mama

Es una enfermedad causada por el crecimiento anormal de células de la mama que forman un tumor maligno

¿Qué tipos de cáncer de mama existen?

In situ
Las células malignas se localizan dentro de los ductos que conducen la leche hasta el pezón

Invasor
Las células malignas crecen fuera de los ductos e invaden otras partes de la mama

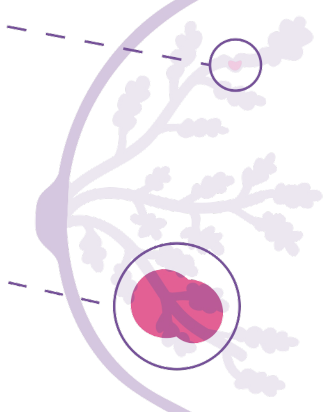

¿Cuáles son las etapas clínicas del cáncer de mama?

Etapa 0: Cáncer in situ.

Etapa 1: Tumor de menos de 2 cm que no afecta los ganglios de la axila.

Etapa 2: Tumor de 2 a 5 cm o que afecta de 0 a 3 ganglios de la axila.

Etapa 3: Tumor mayor a 5 cm o que ha afectado 4 ganglios o más.

Etapa 4: El tumor se ha extendido a otros órganos (cáncer metastásico).

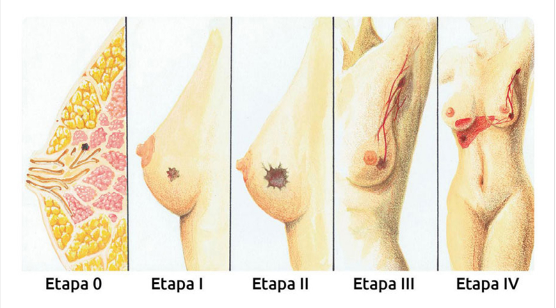

¿Cómo se clasifica el subtipo de cáncer de mama?

La clasificación se basa en los receptores que hacen crecer al tumor.
Las opciones incluyen:

Hormonosensible
El tumor depende de estrógeno o progesterona para su crecimiento.

HER2 postivo
El tumor expresa receptores HER2.

Triple negativo
El tumor no expresa receptores de estrógeno, progesterona o HER2

Conociendo las opciones de tratamiento

Paso 1a: Determinar si es candidata a cirugía de mama.
Existen dos tipos de cirugías disponibles:
- Cirugía conservadora: Se recomienda cuando el tumor es pequeño y está localizado.
- Mastectomia simple o Mastectomia radical: Se utiliza cuando el tumor es más grande o está localizado en dos o más áreas en la misma mama que no están lo suficientemente cercanas para ser extraídas mediante cirugía conservadora.

*Puede acompañarse de reconstrucción mamaria inmediata o tardía.

Paso 1b: Decidir qué tipo de cirugía axilar se necesita.
Existen dos tipos de procedimientos disponibles:
- Ganglio centinela: Se recomienda cuando clinicamente no se aprecia afectación de ganglios linfáticos.
- Disección axilar: Se utiliza cuando hay ganglios linfáticos que parecen estar afectados.

Paso 2: Determinar si se necesita radioterapia.
Se necesita cuando se realiza una cirugía conservadora, el tamaño tumoral es mayor a 5 cm o los ganglios linfáticos se encuentran invadidos.

Paso 3: Evaluar el uso de quimioterapia.
Puede aplicarse antes de la cirugía (en tumores HER2 postivo o triple negativo etapa 2 o cualquier tumor etapa 3) o después del procedimiento quirúrgico.

Paso 4: Considerar tratamiento anti-HER2.
Se requiere cuando el subtipo tumoral es HER2 postivo.

Paso 5: Valorar tratamiento anti-hormonal.
Utilizado cuando el tumor expresa receptores de estrógeno o progesterona.

Paso 6: Analizar si es candidata a recibir inmunoterapia.
Recomendada en casos de cáncer de mama en etapa 4, triple negativo con marcador PD-L1 positivo.

Glosario

Cirugía de mama

Cirugía conservadora:
Se retira el tumor con un margen de tejido sano para dejar el resto de la mama libre de cáncer. Siempre se debe acompañar de radioterapia.

Mastectomía:
Se retira toda la mama.

Cirugía de axila

Ganglio centinela:
Es el primer ganglio al que las células malignas tienen más probabilidad de invadir. Si es negativo, no hay necesidad de quitar el resto de los ganglios de la axila.

Disección radical axilar:
Es un procedimiento donde se quitan los ganglios de la axila cuando están afectados.

Tratamientos

Radioterapia:
Se utiliza radiación en la mama y/o axila para destruir las células malignas y asi reducir las probabilidades de que el cáncer regrese al sitio en donde se originó.

Quimioterapia:
Son medicamentos que se administran por la vena para destruir las células malignas en cualquier parte del cuerpo.

Tratamiento anti-HER2 (trastuzumab y pertuzumab):
Son medicamentos específicos (anticuerpos) que atacan células que tienen receptores HER2 positivos.

Tratamiento anti-hormonal (tamoxifeno, anastrozol, letrozol, exemestano, gosereline, leuprolide o triptoreline):
Son medicamentos que detienen el crecimiento de las células malignas cuando tienen receptores hormonales positivos.

Inmunoterapia (atezolizumab):
Son medicamentos que activan el sistema inmune para atacar al cáncer. Actualmente solo se emplea en el cáncer de mama triple negativo etapa 4 que tiene un receptor especial llamado PD-L1.

Cáncer hereditario

Algunos factores de riesgo para cáncer hereditario:
- Tú o alguien de tu familia fue diagnosticado con cáncer de mama antes de cumplir 50 años.
- Hay varias personas con cáncer de mama y/o de ovario en tu familia.
- Tienes cáncer de mama triple negativo.
- Hay antecedente de otros tipos de cáncer en varios miembros de tu familia.
- Tú o algún familiar tuyo ha tenido cáncer de mama en ambos senos.
- Hay algún hombre en la familia que ha sido diagnosticado con cáncer de mama.

Derechos como paciente

- Tener acceso oportuno a servicios de calidad para diagnóstico y tratamiento.
- Recibir atención médica adecuada, trato digno y respetuoso.
- Recibir información suficiente, clara y veraz.
- Decidir libremente tu tratamiento.
- Contar con facilidades para pedir una segunda opinión.

ALERTA ROSA

@alertarosamx
@alertarosamx
www.alertarosa.com
