# Supplementary material for: Effect of receiving a customizable brochure on breast cancer patients' knowledge about their diagnosis and treatment: A randomized clinical trial
Source: Cancer Med. 2023 Jun 14;12(14):15612–27. doi: 10.1002/cam4.6215 (PMC10417173; doi:10.1002/cam4.6215)
Supplement: Supplementary file 3 — Figure S3. [file CAM4-12-15612-s003.pdf]

KNOWING MY BREAST CANCER

ALERTA ROSA

I am ______________________

We suggest you answer this guide with your doctor to understand your cancer and your treatment options.

Knowing my breast cancer

Breast cancer is a disease caused by the abnormal growth of breast cells that form a malignant tumor.

What type of breast cancer do I have?

- ◯ **In situ**
  The malignant cells are only located inside the ducts that carry milk to the nipple.
- ◯ **Invasive**
  Malignant cells grow outside the ducts and invade other parts of the breast.

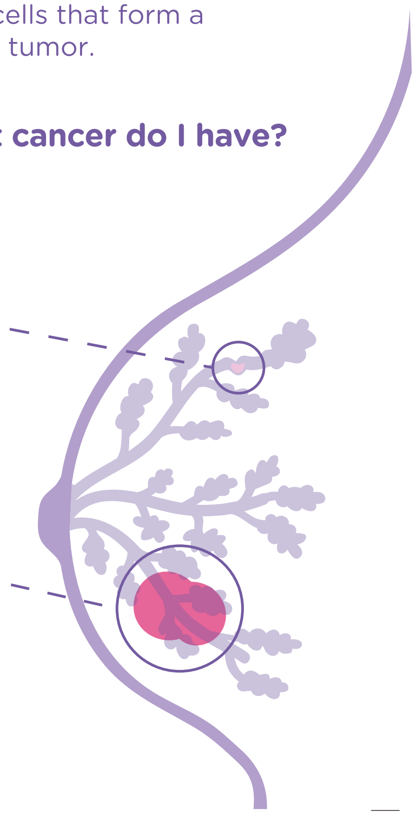

What are breast cancer stages?

- ◯ **Stage 0** — In situ
- ◯ **Stage 1** — The tumor is smaller than 2 cm and has not affected axillary lymph nodes.
- ◯ **Stage 2** — The tumor is 2 to 5 cm and has not affected axillary lymph nodes in the armpit, or has only affected 1 to 3 lymph nodes.
- ◯ **Stage 3** — The tumor is larger than 5 cm, or has affected 4 or more lymph nodes.
- ◯ **Stage 4** — The tumor has spread to other organs (metastatic cancer).

What receptors make my cancer grow?

Estrogen receptor
- ◯ Positive, the tumor depends on estrogen for its growth.
- ◯ Negative.

Progesterone receptor
- ◯ Positive, the tumor depends on progesterone for its growth.
- ◯ Negative.

HER2
- ◯ Positive, the tumor is overloaded with HER2 receptors.
- ◯ Negative.

◯ If all three are negative, my breast cancer is triple-negative.

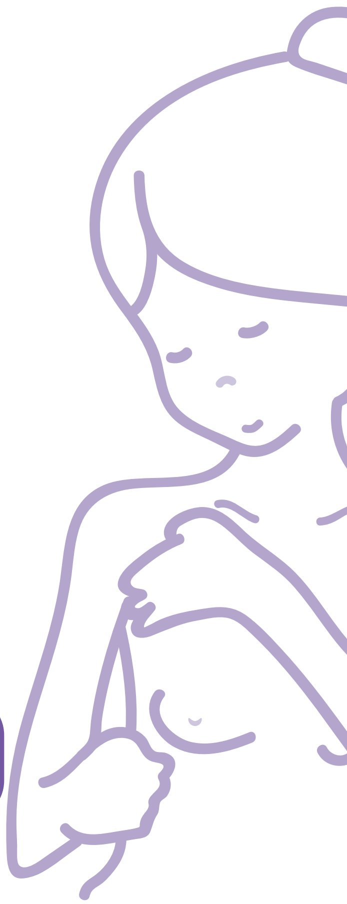

Knowing my treatment options

1. Am I a candidate for breast surgery?
- ◯ Yes
- ◯ No

2. What type of breast surgery do I need?
- ◯ Conservative surgery: My tumor is small and located in only one part of the breast
- ◯ Mastectomy
  - With reconstruction
  - Without reconstruction

3. What type of armpit surgery do I need?
- ◯ Sentinel lymph node biopsy: My nodes seem to be unaffected
- ◯ Radical axillary dissection: My nodes are affected

4. Should I receive radiation therapy?
- ◯ Yes because:
- ◯ No
  - I had conservative surgery
  - My tumor is larger than 5 cm
  - I have positive lymph nodes

5. Am I a candidate for chemotherapy?
- ◯ Yes
- ◯ No
- ◯ Before the surgery:
  - I have stage 3 cancer
  - I have stage 2 cancer and it is HER2 positive or triple-negative
- ◯ After surgery

6. Should I receive anti-HER2 treatment?
- ◯ Yes because:
- ◯ No

My cancer is HER2 positive

7. Am I a candidate for anti-hormonal treatment?
- ◯ Yes because:
- ◯ No

My cancer is estrogen and/or progesterone receptor-positive

8. Should I receive treatment with immunotherapy?
- ◯ Yes because:
- ◯ No

I have stage 4, triple-negative, PD-L1 positive breast cancer

9. Do I need genetic testing to find out if my cancer is hereditary?
- ◯ Yes because:
- ◯ No

I have risk factors for hereditary cancer

10. What complementary services do I require?
- ◯ Genetics
- ◯ Psychology
- ◯ Nutrition
- ◯ Fertility preservation
- ◯ Sexuality counseling
- ◯ Supportive or palliative care
- ◯ Support groups
- ◯ Lymphedema therapy

Glossary

Breast surgery

Conservative surgery:
Only the tumor is removed leaving a margin of healthy tissue and a cancer-free breast. It should always be accompanied by radiotherapy.

Mastectomy:
The entire breast is removed.

Armpit surgery

Sentinel node:
It is the first lymph node that malignant cells are likely to invade. If it is negative, there is no need to remove the rest of the lymph nodes in the armpit.

Radical axillary dissection:
It is a procedure where all the affected lymph nodes in the armpit are removed.

Treatments

Radiotherapy:
Radiation to the breast and/or armpit is used to destroy malignant cells and reduce the chances that breast cancer will return.

Chemotherapy:
These are drugs that are administered through a vein to destroy malignant cells in any part of the body.

Anti-HER2 treatment (trastuzumab and pertuzumab):
These are specific drugs (antibodies) that attack cells that have HER2 receptors.

Anti-hormonal treatment (tamoxifen, anastrozole, letrozole, exemestane, goserelin, leuprolide or triptorelin):
These are drugs that stop the growth of malignant cells when they have positive hormone receptors.

Immunotherapy (atezolizumab):
These are drugs that activate the immune system to attack cancer. Currently, it is only used in stage 4 triple-negative breast cancer with PD-L1 receptors.

Hereditary cancer

Risk factors for hereditary cancer:

- You or someone in your family was diagnosed with breast cancer before their 50th birthday.
- There are several people with breast and/or ovarian cancer in your family.
- You have triple-negative breast cancer.
- There is a history of other types of cancer in several members of your family.
- You or someone in your family has had breast cancer in both breasts.
- There is a biological male in your family who has been diagnosed with breast cancer.

It is important that you know your rights as a patient

- Access to timely high-quality services for diagnosis and treatment.
- Adequate medical care, and dignified and respectful treatment.
- To receive sufficient, clear, and truthful information.
- To freely choose your treatment.
- To request a second opinion.

Claim your rights!

ALERTA ROSA
@alertarosaapp
@alertarosamx
www.alertarosa.com
